# Supplementary material for: Empirical methods for controlling false positives and estimating confidence in ChIP-Seq peaks
Source: BMC Bioinformatics. 2008 Dec 5;9:523. doi: 10.1186/1471-2105-9-523 (PMC2628906; doi:10.1186/1471-2105-9-523)
Supplement: Additional File 1 — A variety of html documents from the USeq web site detailing the available applications, their best usage, output file type descriptions, command line menus, etc. [file 1471-2105-9-523-S1.zip › USeqUserGuides/JavaDoc.zip!/JavaDoc/util/gen/PearsonCorrelation.html]

PearsonCorrelation


|  |  |  |
| --- | --- | --- |
| **Overview** \| **Package** \| **Class** \| **Use** \| **Tree** \| **Deprecated** \| **Index** \| **Help** | |  |
| **PREV CLASS**   **NEXT CLASS** | **FRAMES**    **NO FRAMES**     **All Classes** |  |
| SUMMARY: NESTED \| FIELD \| CONSTR \| METHOD | DETAIL: FIELD \| CONSTR \| METHOD |  |


---


## util.gen Class PearsonCorrelation

```
java.lang.Object
  util.gen.PearsonCorrelation
```

---

``` public class PearsonCorrelation extends java.lang.Object ```

Calculates Pearson Correlations (R) in two ways, using a straight static method on two arrays or
by 1st adding paired arrays sequentially and 2nd calculating the correlation on the cumulative
sum. This later method is good if you want to keep the memory requirements down. Otherwise
just use the static methods.

---

**Constructor Summary**

`PearsonCorrelation()`


| **Method Summary** | |
| --- | --- |
| `void` | `addPairsToCorrelate(float[] x, float[] y)`             Use this method to sequentially add pairs for correlation. |
| `java.lang.Double` | `calculateAdditivePairCorrelation()`             Following loading of this class using the addPairsToCorrelate(), call this method to calculate to correlation coefficient (R) on the total. |
| `static double` | `correlationCoefficient(double[] x, double[] y)`             Calculates Pearson correlation coefficient, r, from two double[]s |
| `static double` | `correlationCoefficient(float[] x, float[] y)`             Calculates Pearson correlation coefficient, r, from two float[]s. |

**Methods inherited from class java.lang.Object**

`equals, getClass, hashCode, notify, notifyAll, toString, wait, wait, wait`

**Constructor Detail**

### PearsonCorrelation

```
public PearsonCorrelation()
```


**Method Detail**

### addPairsToCorrelate

```
public void addPairsToCorrelate(float[] x,
                                float[] y)
```

:   Use this method to sequentially add pairs for correlation.

---


### calculateAdditivePairCorrelation

```
public java.lang.Double calculateAdditivePairCorrelation()
```

:   Following loading of this class using the addPairsToCorrelate(),
    call this method to calculate to correlation coefficient (R) on the total.
    Returns null if an error is encountered.

---


### correlationCoefficient

```
public static double correlationCoefficient(float[] x,
                                            float[] y)
```

:   Calculates Pearson correlation coefficient, r, from two float[]s.
    Cannot have one float[] be uniform values, returns -2 if error.

---


### correlationCoefficient

```
public static double correlationCoefficient(double[] x,
                                            double[] y)
```

:   Calculates Pearson correlation coefficient, r, from two double[]s


---


|  |  |  |
| --- | --- | --- |
| **Overview** \| **Package** \| **Class** \| **Use** \| **Tree** \| **Deprecated** \| **Index** \| **Help** | |  |
| **PREV CLASS**   **NEXT CLASS** | **FRAMES**    **NO FRAMES**     **All Classes** |  |
| SUMMARY: NESTED \| FIELD \| CONSTR \| METHOD | DETAIL: FIELD \| CONSTR \| METHOD |  |


---
